# Diastolic Cardiac Function by MRI—Imaging Capabilities and Clinical Applications

**DOI:** 10.3390/tomography7040075

**Published:** 2021-12-08

**Authors:** El-Sayed H. Ibrahim, Jennifer Dennison, Luba Frank, Jadranka Stojanovska

**Affiliations:** 1 Department of Radiology, Medical College of Wisconsin, Milwaukee, WI 53226, USA; lufrank@mcw.edu; 2 Department of Medicine, Medical College of Wisconsin, Wausau, WI 54401, USA; jdennison@mcw.edu; 3 Department of Radiology, New York University, New York, NY 10003, USA; Jadranka.Stojanovska@nyulangone.org

**Keywords:** heart, diastolic cardiac function, MRI, HFpEF, strain, cardiac

## Abstract

Most cardiac studies focus on evaluating left ventricular (LV) systolic function. However, the assessment of diastolic cardiac function is becoming more appreciated, especially with the increasing prevalence of pathologies associated with diastolic dysfunction like heart failure with preserved ejection fraction (HFpEF). Diastolic dysfunction is an indication of abnormal mechanical properties of the myocardium, characterized by slow or delayed myocardial relaxation, abnormal LV distensibility, and/or impaired LV filling. Diastolic dysfunction has been shown to be associated with age and other cardiovascular risk factors such as hypertension and diabetes mellitus. In this context, cardiac magnetic resonance imaging (MRI) has the capability for differentiating between normal and abnormal myocardial relaxation patterns, and therefore offers the prospect of early detection of diastolic dysfunction. Although diastolic cardiac function can be assessed from the ratio between early and atrial filling peaks (E/A ratio), measuring different parameters of heart contractility during diastole allows for evaluating spatial and temporal patterns of cardiac function with the potential for illustrating subtle changes related to age, gender, or other differences among different patient populations. In this article, we review different MRI techniques for evaluating diastolic function along with clinical applications and findings in different heart diseases.

## 1. Introduction

The left ventricle (LV) of the heart fills via two separate mechanisms. In early-diastole, the LV fills passively through active relaxation. In late-diastole, the remaining blood contributing to the total end-diastolic volume enters the ventricle via active contraction of the left atrium (LA). During isovolumetric relaxation, LV volume increases due to alterations in principal strains and untwisting of the ventricles [1]. Complete appreciation of impaired diastolic function as it relates to cardiovascular disease requires detailed analysis of myocardial tissue deformation during diastole, e.g., using magnetic resonance imaging (MRI) strain imaging. While diastolic shear strain rates are coupled to the prior systolic shear strain constituents, torsional recoil is independent of end-systolic factors [2].

Diastolic dysfunction is an indication of abnormal mechanical properties of the myocardium, characterized by slow or delayed myocardial relaxation, abnormal LV distensibility, and/or impaired LV filling [3]. Diastolic dysfunction has been shown to be associated with age and other cardiovascular risk factors such as hypertension and diabetes mellitus [4]. In addition, several cardiovascular diseases cause adverse LV remodeling, which leads to diastolic dysfunction. Especially, heart failure with preserved ejection fraction (HFpEF) is a clinical syndrome where patients have normal LV systolic function, and evidence of diastolic dysfunction [5]. HFpEF has distinct causes and differential pathophysiology from those with systolic heart failure and nearly 50% of patients presenting with symptoms of heart failure (HF) have diastolic dysfunction. HFpEF is more prevalent than heart failure with reduced ejection fraction (HFrEF) among women and in those with elevated systemic blood pressure [6]. Additionally, patients presenting with HFpEF suffer from vascular changes and ventricular remodeling that could affect the physiological relationships between afterload and diastole and cause ischemia induced by supply-demand imbalance [7]. Furthermore, HFpEF is correlated to substantial morbidity and mortality. Unfortunately, accurate non-invasive diagnosis of LV diastolic dysfunction remains difficult, leading to challenges in the diagnosis and treatment of HFpEF [8].

Heart catheterization is the current gold standard for demonstrating the characteristics of diastolic heart failure, but due to the risks and costs involved with invasive hemodynamic evaluation, it is not practical for the diagnosis of diastolic dysfunction. Echocardiography, with significantly less risk compared to heart catheterization, is currently the method of choice for diagnosing diastolic dysfunction. Nevertheless, echocardiography has limitations related to poor acoustic windows, geometric assumptions, suboptimal spatial resolution, and high dependency on the operator’s skills. Some of these limitations restrict the technique’s ability to accurately measure annular velocities and assess diastolic function if regional dysfunction is present [9]. Cardiac MRI measurements, however, are superior to echocardiography in accuracy for the evaluation of diastolic function. This makes cardiac MRI a valuable imaging modality for the analysis of diastolic function in patients with cardiovascular diseases such as hypertrophic cardiomyopathy, hypertension, aortic valve stenosis, coronary artery disease, and congestive heart failure [10]. Regarding this review, the authors conducted an advanced search of the PubMed database using the following keywords: cardiac/heart, MRI/magnetic resonance, diastole/diastolic, which resulted in >40 papers that are covered in this comprehensive review, where different studies are grouped based on cardiovascular diseases and implemented techniques. Table 1 summarizes key studies in which MRI was used for evaluating diastolic heart function [10].

## 2. Cardiac MRI Techniques

Different MRI techniques are currently available for evaluation of diastolic cardiac function, such as cine imaging for global function analysis, phase contrast (PC) imaging for flow analysis, and myocardial tagging for regional function analysis. Simple measurements, such as longitudinal fractional shortening, can be measured quickly and easily by MRI, which has reliably identified echocardiography-evidenced diastolic dysfunction in patients with preserved LV EF [5]. Assessment of myocardial contractility pattern using other MRI methods, such as strain-encoded (SENC), displacement encoding with stimulated echoes (DENSE), and MRI feature-tracking (MRI-FT), contribute to further evaluation of regional diastolic function. Moreover, recent advances in MRI techniques have enabled non-invasive assessment of vascular compliance and elastic properties of the vessel wall using pulse wave velocity (PWV) measurements, PC MRI, and MRI tagging. ^31^P magnetic resonance spectroscopy (MRS) is another technique for non-invasively quantifying the energy required for active relaxation of the myocardial tissue through calculating the ratio of myocardial phosphocreatine to adenosine triphosphate (PCr/ATP).

**Figure 1:**
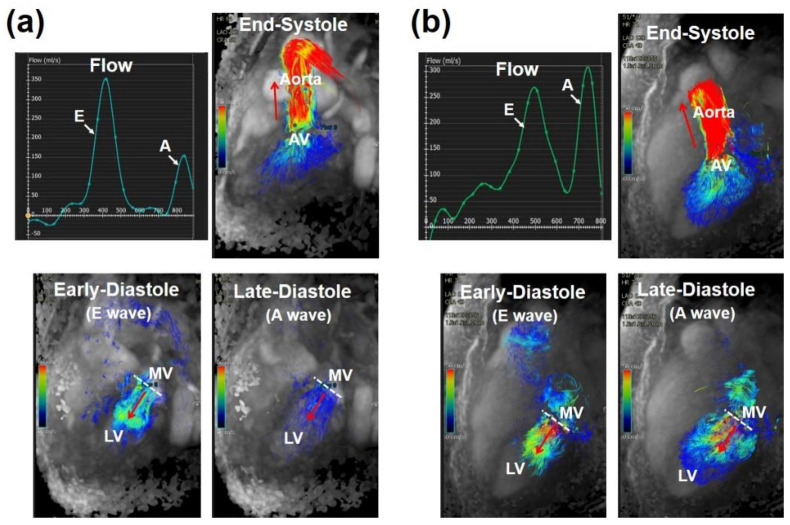
Mitral flow analysis in (**a**) normal case and (**b**) diastolic dysfunction, showing early (E) and atrial (A) filling peaks. E/A ratio shows normal (>1) and abnormal (<1) diastolic function in normal and pathological cases, respectively. Four-dimensional (4D) flow images are shown at three timepoints in the cardiac cycle (early systole, early diastole, and late diastole), confirming the analysis findings (E & A filling peaks). White dotted line shows mitral valve plane and red arrow shows flow direction. LV, left ventricle; MV, mitral valve.

Global diastolic function is assessed using time-volume curves generated by tracing the epicardial and endocardial borders of the myocardium in cine MRI images throughout the cardiac cycle or from 4D flow images across atrioventricular valves (Figure 1). Studies are undergoing to create methods that would allow for efficient assessment of the diastolic cardiac function. For example, Young et al. [28] developed a three-dimensional (3D) model of cardiac function based on standard cine MRI images and showed that the developed model is equally capable of identifying diastolic dysfunction as echocardiography. The authors showed that the most useful MRI parameters for assessing LV diastolic function are E/E’ (the ratio of early-peak filling rate to early-longitudinal relaxation rate), NE (normalized early-peak filling rate, defined by early-peak filling rate divided by end-diastolic volume), and E/A (the ratio of early-peak filling rate to atrial-peak filling rate). The categorization of diastolic dysfunction was accomplished using septal and lateral measurements obtained to evaluate longitudinal shortening [29].

### 2.1. MRI Tagging

**Figure 2:**
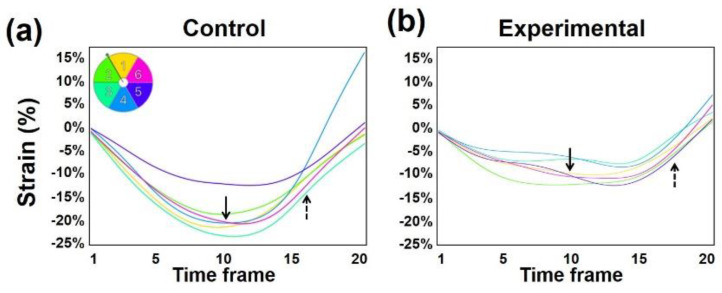
Reduced left ventricle myocardial contractility in a rat model of lung cancer radiation therapy (experimental). Segmental circumferential strain curves in six segments (color code shown on top left) in a mid-ventricular short-axis slice in a normal rat (**a**) and an experimental rat (**b**) that received localized heart irradiation of 24 Gy. Note reduced peak systolic strain (solid arrows) and diastolic strain rate (dotted arrows) post radiation.

The heart function can also be studied using MRI tagging [30]. MRI tagging has advantages for evaluating diastolic heart function through excellent soft-tissue contrast and the ability to directly measure myocardial relaxation (Figure 2), in contrast to Doppler echocardiography which is load dependent and limited to indirect measures of LV function [31]. Ambale-Venkatesh et al. [32] used MRI tagging to quantify end-diastolic strain rate and strain relaxation index to predict the development of HF in patients with no prior history of cardiovascular disease. The authors used a novel index of diastolic function, the strain relaxation index, which factors in both the interval between the occurrence of peak systolic strain and post-systolic peak as well as the early-diastolic strain rate.

### 2.2. Complementary Spatial Modulation of Magnetization

The introduction of complementary spatial modulation of magnetization (CSPAMM) by Fischer et al. [33] helped resolve the issue of tagline fading later in the cardiac cycle in conventional tagging, and therefore allowed for analysis of heart contractility during late-diastole. CSPAMM tagging has been shown to be a reliable method for the analysis of cardiac wall motion, strain, and twist [34]. Many studies have been developed around CSPAMM to measure different parameters of global and regional ventricular function and contractility and to study the effects of aging, ischemic and structural heart diseases, and cardiomyopathies on regional cardiac function [35]. CSPAMM tagging has been used for the evaluation of hypertrophic cardiomyopathy, where it showed alterations of total systolic shortening and diastolic strain rates as well as the overlap of apical untwisting with LV filling [36,37]. Other studies used CSPAMM to evaluate the effects of aortic stenosis (AS) on ventricular function [15,18] and recovery of cardiac function following transcatheter aortic repair [38].

### 2.3. MRI Feature-Tracking

MRI feature-tracking (MRI-FT) is yet another technique for evaluating regional cardiac function directly from the cine images [30]. Ng et al. [39] analyzed MRI-FT-based strain parameters for their ability to identify diastolic dysfunction and assess their correlation with echocardiography indices. The results showed that LV circumferential diastolic strain rate is able to detect diastolic dysfunction with results similar to those obtained by echocardiography.

### 2.4. Phase-Contrast MRI

**Figure 3:**
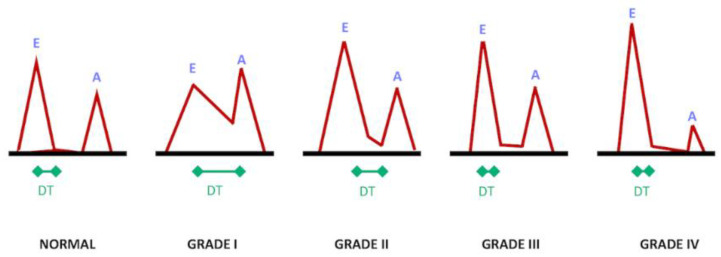
Grades of diastolic dysfunction (I–IV) based on trans-mitral flow pattern. E, early-diastolic flow; A, atrial/late-diastolic flow; DT, deceleration time.

Phase-contrast (PC) MRI offers an alternative method to echocardiography for assessing vascular blood flow and myocardial tissue velocity. PC MRI has the potential to measure hemodynamic parameters that are important for assessing diastolic function in clinical routine [40] (Figure 3), with investigational studies showing promise for measuring pressure gradients. PC MRI velocity indices of diastolic function have been shown to correlate with corresponding measurements obtained by Doppler echocardiography [41]. Ashrafpoor et al. [42] demonstrated the capability of PC MRI for independently characterizing subclinical age-related variations in diastolic function among healthy volunteers. The results showed that LV remodeling index and global myocardial wall thickness have a strong correlation with diastolic parameters related to LV and myocardial relaxation, such as myocardial longitudinal peak velocity, deceleration time, and isovolumetric relaxation time [42].

### 2.5. Four-Dimensional Flow Cardiac MRI (4D-Flow)

Four-dimensional flow (4D-Flow) MRI is superior to Doppler echocardiography for evaluating intracardiac velocity as it is not limited by flow direction or inconsistency in transducer alignment. Previous studies using 4D-Flow MRI demonstrated significant flow reduction and end-diastolic volume and kinetic energy increase in patients with moderate-to-severe dilated cardiomyopathy [43]. The residual volume at end-diastole can be divided into four functional flow components: direct flow (blood that enters and exits the LV within the same cycle), retained inflow (blood that enters the LV but does not exit during the same cycle), delayed ejection flow (blood that had remained in the LV from the cycle before and is ejected during the current cycle), and residual volume (blood that is stagnant in the LV, not entering nor exiting during the cycle). The division of LV end-diastolic volume in this way showed that direct flow diminishes as LV volume increases and that non-ejecting volume contributing to LV end-diastolic volume (EDV) increases in patients with normal-to-mild LV remodeling and normal-to-mildly depressed LV systolic function [44].

## 3. Cardiovascular Measures

### 3.1. Temporal Resolution

While a low temporal resolution does not seem to affect accuracy in the calculation of LV systolic function, it does affect rate-based indices of LV diastolic function_,_ resulting in underestimation of the absolute volume change of respective indices [45]. Several attempts have been made to improve MRI methods for measuring blood or tissue velocities in LV diastolic analysis, although there are some trade-offs in terms of limited quality, poor temporal resolution, long acquisition times, or sophisticated post-processing methods. By using a respiratory-triggered free-breathing cine sequence, Zhang et al. [45] achieved high temporal resolution that has the potential for evaluation of both LV systolic and diastolic functions from a single stack of cine MRI data.

### 3.2. Late Diastole

Understanding normal variants and pathological differences in diastolic function could be achieved through detailed study embodying the relationship between early- and late-diastolic measurements [46]. While the majority of LV filling occurs at early-diastole in normal physiological conditions, in the presence of diastolic dysfunction, impaired LV relaxation causes a shift of LV filling into late-diastole, associated with LA systolic function. MRI offers promise for early detection of diastolic dysfunction through its ability to distinguish normal from abnormal diastolic patterns. While it has been used to demonstrate characteristics of early-diastole [47], little work has been carried out to study the cardiac function in late-diastole. For example, tag lines fading toward the end of the cardiac cycle limits the usefulness of conventional MRI tagging for studying late-diastolic cardiac function [46]. However, the recent development of MRI-FT techniques offers an alternative option to study myocardial contractility during the late-diastolic phase.

### 3.3. Untwisting Motion

The processes of myocardial contraction and relaxation during the cardiac cycle are complex, yet organized sequences of events, which under normal function create conditions for maximal cardiac output. During systole the apex and base twist in opposite directions. At the same time, both ventricles contract to pull the atrioventricular plane toward the apex. Diastole is initiated by an instantaneous untwisting of the apex where a change in chamber volume and shape is negligible, followed by relaxation and untwisting of the remaining myocardium resulting in passive filling of the ventricle [35]. There are three components of myocardial relaxation that occur before the aortic valve closes, which produce an abrupt decline in LV pressure that initiates early filling of the LV: (1) release of torsion; (2) shear strain; and (3) radial thinning. Following aortic valve closure, and before the opening of the mitral valve, there is zero change in actual LV volume despite the seemingly increase in ventricular size observed due to distension of the LV myocardium.

Analysis of systolic and diastolic function has been conducted by quantification of torsion and recoil rate, respectively [48]. Kowallick et al. [49] demonstrated that increases in subendocardial torsion and global recoil rate coincide with increasing doses of dobutamine. The authors used MRI-FT indices, which showed to be accurate and reproducible means of quantifying myocardial torsion and recoil rates both at rest and stress. In another study, Dorfman et al. [50] demonstrated impaired diastolic untwisting in a cohort of adults after a period of post-exercise rest. Using MRI tagging to study the effect of inactive lifestyle on myocardial untwisting rate, they demonstrated a notable decline in untwisting rate in the mid-wall, slightly less significant reduction in the endocardium, and no alteration in the epicardium. The authors also showed that a lack of cardiovascular exercise leads to reduced LV mass and end-diastolic volume. 

Distinct perturbations of diastolic untwisting, as demonstrated by MRI tagging, helped differentiate physiologic from pathologic hypertrophy [18]. The characteristics of ventricular rotation with clear distinction of early apical untwisting from the onset of ventricular filling showed to be the same in normal healthy hearts and hearts of endurance athletes with physiologic hypertrophy [15]. Contrarily, in patients who develop hypertrophy due to pathologic conditions such as overload or aortic stenosis, the velocity of apical rotation at end-systole is increased and the time to maximum velocity of apical untwisting is delayed, resulting in concurrent relaxation of the apex and filling of the ventricle [15]. Interruption of the normal occurrence of apical untwisting is also seen in patients with myocardial infarction (MI) [51]. However, in this case, there is no separation of apical untwisting from LV filling, and the peak velocity of apical rotation is remarkably reduced.

### 3.4. Vorticity

The presence of diastolic vortices within the LV contributes to the transfer of fluid kinetic energy between cardiac chambers, which indicates the presence of healthy ventricular function [52]. Decreased LV vortex formation has been demonstrated in patients with both dilated and hypertrophic cardiomyopathies [53] and in HF [54].

### 3.5. Left Atrium

Left atrial (LA) remodeling can occur in the setting of HF; however, little is known about the pathophysiology behind this phenomenon. Seemann et al. [55] evaluated multiple parameters of cardiac diastolic function using cine and PC MRI and demonstrated a correlation between atrial fibrosis and diastolic dysfunction. In another study, Aquaro et al. [56] showed that alteration of LA contractility parameters was present before that in the LV in diastolic dysfunction. The authors were able to create a diagnostic algorithm for diastolic dysfunction, which includes multiple calculations using measurements of LA and LV volumes, including atrial emptying fraction, isovolumetric pulmonary vein transit volume, and isovolumetric pulmonary vein transit ratio. The results confirmed that LA plays a significant role in the evolution of diastolic dysfunction and that reliable grading of diastolic dysfunction can be achieved by cardiac MRI analysis of both left-sided chambers of the heart. More recently, Kermer et al. [8] used MRI to assess cardiac structure and function in order to detect diastolic dysfunction by assessing the LV and LA functions through using tissue tracking, tagging, tissue phase mapping, and PC sequences and comparing the results to published gold standards including invasive measurements. The results showed that the MRI techniques used to calculate enlarged LA dimensions have high diagnostic accuracy and are predictive for identifying diastolic dysfunction. The results also showed that the impaired contractility pattern of the basal lateral wall is a direct sign of diastolic dysfunction.

### 3.6. Mitral Annulus

Not much data exists on mitral annular motion as it relates to diastolic function [40]. In one study, Wu et al. [9] used long-axis cine MRI images for mitral annular analysis using 3D mitral annular sweep volumes to determine parameters capable of detecting diastolic dysfunction. Three-dimensional (3D) volume tracking of the mitral annulus showed superiority over other methods such as MRI tagging that cannot evaluate atrial systole with the same reliability [9]. The reversal of the ratio of peak sweep rate in early-diastole to peak sweep rate in atrial systole could possibly be explained by LA dilation and subsequent enhancement of atrial contraction in concordance with the Frank–Starling law [9,57].

## 4. Heart Failure with Preserved Ejection Fraction

Though factors affecting HFpEF are not well understood, Edvardsen et al. [31] reported that HFpEF may begin as a regional process in the same way as HFrEF. The capability of MRI for generating multiple parameters about global and regional cardiac functions and myocardial tissue characterization makes it a valuable tool for evaluating HFpEF in clinical trials. For example, as part of the Multi-Ethnic Study of Atherosclerosis (MESA) clinical trial, Edvardsen et al. [31] used MRI to study regional LV diastolic function in asymptomatic HFpEF patients and found a reduction of regional diastolic function by approximately 30% in the patients regardless of age or sex. In another study on 1582 subjects from the MESA trial, Ladeiras-Lopes et al. [58] used cardiac MRI to evaluate the association of metabolic syndrome with diastolic function and myocardial extracellular matrix. The results showed that adults without diabetes with metabolic syndrome, as well as patients with diabetes, have impaired diastolic function irrespective of the myocardial interstitium. In a third study on a smaller cohort of the MESA trial, Nacif et al. [29] used MRI to develop and validate a three-dimensional model-based volumetric assessment of diastolic function and compared the results to those obtained with echocardiography, where the authors showed that the developed model is able to identify study subjects with reduced diastolic function and showed good reproducibility. Recently, Mordi et al. [59] studied a cohort of 112 subjects and showed that cardiac MRI can differentiate among HFpEF patients, hypertensive patients, and healthy control subjects. 

MRI tagging enables longitudinal monitoring of myocardial relaxation and diastolic function in various clinical conditions. For example, MRI tagging can classify those at risk of developing HFpEF [30]. One study showed that circumferential strain is a strong predictor of HF in a cohort of subjects without clinical evidence of cardiovascular diseases, irrespective of age, diabetes, hypertension, MI, and LV EF [60]. MRI-FT has been used to investigate the prevalence and characteristics of global longitudinal strain impairment in a cohort with HFpEF and the results were compared to those obtained by LV cardiac catheterization in the same patients [61]. Ito et al. [61] established that global longitudinal strain obtained using MRI-FT has an independent association with LV diastolic time constant, Tau. In consideration of other reports that showed LV stiffness and delayed relaxation as the major causes for diastolic dysfunction [62], it is intuitive to say that these findings support the affiliation of systolic longitudinal dysfunction and diastolic dysfunction in HFpEF [61]. The coupling of LV contraction and relaxation has been previously illustrated [63]. That is, a reduction in LV contractility correlates to a reduction in the rate of LV relaxation in patients with HFpEF. It has been shown that a decrease in endocardial function leads to decreased global longitudinal strain, which is compensated for by global circumferential strain and LV twisting in patients with HFpEF [64]. 

**Figure 4:**
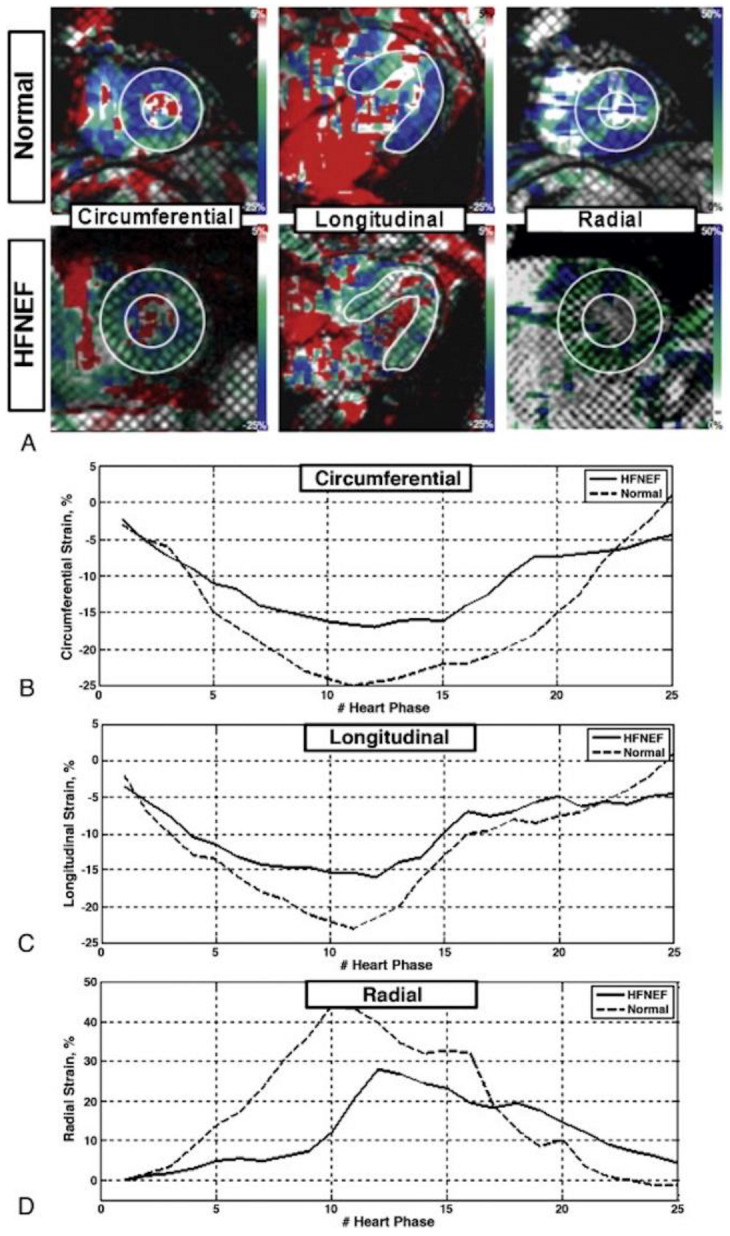
Myocardial strain in heart failure with normal ejection fraction (HFNEF) and normals. (**A**) Representative color-coded myocardial strain maps at end-systole from a volunteer (up) and HFNEF patient (down). Left, middle, and right panels show circumferential, longitudinal, and radial strains, respectively. Representative circumferential (**B**), longitudinal (**C**) and radial (**D**) strain curves are shown from normal (dashed line) and HFNEF (solid line) cases during the cardiac cycle. The results show higher dynamic strain range (difference between end-systolic and end-diastolic strains) in normal compared to HFNEF, which explains the less relaxation experienced in HFNEF during diastole. Figure reproduced with permission from [67]. Copyright 2011 Elsevier.

MRI-generated measurements of diastolic cardiac function have been compared to those from echocardiography and tissue Doppler imaging (TDI) in a number of studies. For example, in a study by Buss et al. [40], the authors showed that measurements from phase-contrast MRI correlated with TDI regarding the relation of mitral E and A velocities (R = 0.83, *p* < 0.001). In the same year, Wu et al. [9] showed that MRI-derived diastolic parameters were effective in identifying patients with diastolic dysfunction when correlated with TDI-based variables. This was confirmed in a more recent study by Nacif et al. [29] who demonstrated that the E/A ratios from TDI were positively associated with E/A ratios from MRI (R = 0.71, *p* < 0.0001) with a small bias (0.081%) toward a higher E/A ratio by MRI. In a similar study conducted in the same year, Seemann et al. [55] showed that the ratios E/A and E/e’ by MRI and TDI have a strong agreement (R = 0.80, *p* = 0.0006 for E/A and R = 0.85, *p* = 0.0004 for E/e’).

van Heerebeek et al. [65] proposed that it is not fibrosis, but myocyte stiffness, that is indicated as the primary contributor to diastolic dysfunction in diabetic patients suffering from HFpEF. However, it has been demonstrated that postcontrast T1 time can independently predict myocardial stiffness as a consequence of an increase in extracellular matrix, which allowed for establishing a link between fibrosis and myocardial stiffness [61,62,66]. The finding that myocardial fibrosis, as assessed by T1-mapping-based extracellular volume (ECV), correlates with myocardial stiffness could be useful for identifying the mechanism of diastolic dysfunction in patients with HFpEF [62]. In this regard, further research is needed to establish the role of T1 and ECV mappings in clinical management of patients with diastolic dysfunction as in HFpEF. In their study of eight patients with HFpEF and eight controls using MRI tagging, Ibrahim et al. [67] found that, compared to controls, patients with HFpEF had decreased LV filling rate, peak strain, and early-diastolic strain rate, and reversal of early-to-atrial (E/A) filling ratio (Figure 4). The authors analyzed the relationship between several parameters of LV function and the ability of the aorta to buffer changes in systemic blood pressure in patients with heart HFpEF. The authors showed the presence of an inverse relationship between a reduced E/A ratio and myocardial strain rate, and implicated aortic atherosclerosis as a major contributor to HFpEF. Table 2 shows differences in ventricular parameters in different groups of diastolic dysfunction [56].

## 5. Effectors of Diastolic Function

### 5.1. Aging

Age is one of the different factors that can affect diastolic function. It has been indicated that 30–50% of elderly subjects with heart failure have an apparently preserved systolic function as assessed by EF. The risk of a compromised cardiac reserve is indicated by the association between cardiac remodeling and diastolic function in the elderly. Recently, Lin et al. [68] used heart deformation analysis indices derived from cine MRI to distinguish age-related changes in LV wall motion during early and late diastole. The results showed that, in comparison to younger subjects, the effects of aging caused lower displacement, velocity, and strain rates in early diastole, but higher peak circumferential strain rates in late diastole. The authors, therefore, suggested that reduced LV compliance with a possible compensatory increase in active relaxation is associated with aging. Multiple other effects of aging on diastolic function have been reported in a limited number of studies, such as reduction and prolongation of diastolic longitudinal and radial velocities [69], reduced and prolonged longitudinal and radial velocities during diastole [70], reduction of peak rates of circumferential and longitudinal relaxations and torsion reversal, and increase of regional asynchrony in time-to-peak rates of circumferential and longitudinal relaxations [71]. Moreover, age was demonstrated to have a strong independent association with peak myocardial velocity and flow-rate parameters [42]. Likewise, and in line with the reduction in diastolic function due to aging, Ambale-Venkatesh et al. [32] showed a temporal decrease in early diastolic strain rate with a concurrent increase in strain relaxation index, which predicted heart failure and atrial fibrillation independent of other cardiovascular disease confounders.

Other notable age-related differences in ventricular function were reported by Foll et al. [70], who used tissue phase mapping to show faster diastolic radial velocities than systolic radial velocities in young subjects and higher longitudinal velocities in younger women [70]. Conversely, older women were found to have a regional reduction in longitudinal velocities as well as prolonged time-to-peak apical longitudinal velocities throughout the cardiac cycle compared to older men [70]. In another study, Ashrafpoor et al. [42] showed that a precise longitudinal assessment of LV diastolic function could be useful for predicting the onset of HF and distinguishing pathological diastolic dysfunction from age-related effects. Furthermore, the authors reported a comprehensive evaluation of age-related variation in diastolic function parameters with corresponding reference ranges, where this data were able to characterize subclinical age-related variations in LV diastolic function in healthy individuals. 

In a cardiac MRI study of right ventricular (RV) function [72], a notable reduction in the absolute and normalized early peak filling rates and elevation of the absolute and normalized active peak filling rates were found in adult males as compared to adult females. Another important difference for the evaluation of RV structure and function was found between men and women in a study of RV volume in which men were found to have much higher RV volume than women [73].

### 5.2. Diabetes

Diabetes is another common condition that affects the heart. Associated cardiovascular complications such as coronary artery disease and LV dysfunction are prevalent among patients with diabetes mellitus type 2, and therefore, diabetic patients succumb to cardiovascular disease more than the general population [74]. Furthermore, signs of diastolic impairment can occur before symptoms of LV dysfunction occur in patients with type 2 diabetes [75]. Here, the underlying pathophysiology leading to diastolic dysfunction in diabetic patients could be closely related to abnormal myocardial perfusion [76] and/or macrovascular coronary artery disease leading to distal embolization and microinfarctions [77]. Graca et al. [74] investigated the possible influence of coronary artery disease on LV diastolic function by obtaining coronary artery calcification scores by CT, and comparing cardiac MRI-derived parameters between patients with uncomplicated type 2 diabetes mellitus and control subjects. The results showed that patients with diabetes mellitus type 2 and coronary artery disease have more LV diastolic impairment than those without coronary artery disease. Thus, this data supports the hypothesis that in the presence of coronary artery disease, diastolic function is compromised in diabetic patients free of LV dysfunction symptoms.

Vascular complications due to type 1 diabetes mellitus have been shown to lead to structural changes and subsequent stiffening of the aortic wall [78]. Furthermore, the association of hypertension and advanced diabetic heart disease with diastolic dysfunction can be largely attributed to myocardial fibrosis causing cardiac remodeling [79]. In their study of patients with type 1 diabetes mellitus, van Shinkel et al. [80] used aortic PWV and speckle tracking strain analysis to assess the involvement of aortic stiffness with sub-clinical LV diastolic dysfunction and LA compliance, respectively. They found that as aortic stiffness increases, LV diastolic function and LA compliance decrease in patients with diabetes mellitus type 1 [80]. Specifically, the data showed that aortic pulse wave velocity correlations with LV diastolic function and LA strain measurements were not age dependent in type 1 diabetes mellitus. LA strain has previously been understood to be dependent on LV systolic function; however, subjects in this study had preserved LV systolic function, making it the first study to demonstrate a strong association between aortic stiffness and LA strain [80]. High end-diastolic pressure and increased afterload is one possible mechanism responsible for the correlation between aortic stiffness and LV diastolic dysfunction [81]. Furthermore, cross-linked collagen molecules, due to advanced glycation products formed in the presence of diabetes mellitus, cause changes in both myocardium and vessel walls [82].

### 5.3. Metabolic Syndrome

Ladieras-Lopes et al. [58] evaluated the association of metabolic syndrome with diastolic function and myocardial extracellular matrix using cardiac MRI in order to better understand the effects of metabolic syndrome and insulin resistance on diastolic dysfunction and myocardial fibrosis. Resulting data showed that metabolic syndrome is associated with impaired diastole even in the absence of type 2 diabetes due to intrinsic cardiomyocyte alterations unrelated to interstitial disease [58] supporting the idea of ’insulin-resistant’ cardiomyopathy and associated pathophysiologic changes including myocardial metabolic deregulation, oxidative stress, and inflammation [83]. Insulin resistance also leads to cellular injury and changes in contractile proteins, calcium load, and the sympathetic nervous system, possibly contributing to myocardial stiffness without an increase in the extracellular matrix as extracellular volume quantification using cardiac MRI did not show an association with increased extracellular matrix in metabolic syndrome or type 2 diabetes [58]. In addition, the pathophysiologic changes in metabolic syndrome cause an increased risk of cardiovascular disease which can then lead to remodeling and dysfunction and ultimately ‘insulin-resistant’ cardiomyopathy [83].

## 6. Coronary Artery Disease

LV diastolic dysfunction can be precipitated by the same aggravating factors that play a role in the development of atherosclerosis, either primarily (due to hypertension or age-related reduction in vascular compliance) or secondarily (due to ischemia-related changes in the myocardium) [77]. Despite these known relations, data published on the relationship between coronary artery disease and LV diastolic function using cardiac MRI methods are limited and controversial [31,84,85]. Nevertheless, the question that remains to be answered is which insult promotes the development of HFpEF: atherosclerosis, myocardial fibrosis, or a combination of the two [67].

### 6.1. Acute Coronary Syndrome

Acute coronary syndrome causes rapid onset of LV diastolic dysfunction, where early detection of impaired function is useful for appropriate care management [86]. Unfortunately under current guidelines, 75% or more of patients who present with chest pain are not accurately diagnosed with acute coronary syndrome [87] with nearly 10% of those resulting in eventual myocardial infarction [88]. Additionally, post-acute coronary syndrome has been related to poorer prognosis and higher risk of mortality [89].

**Figure 5:**
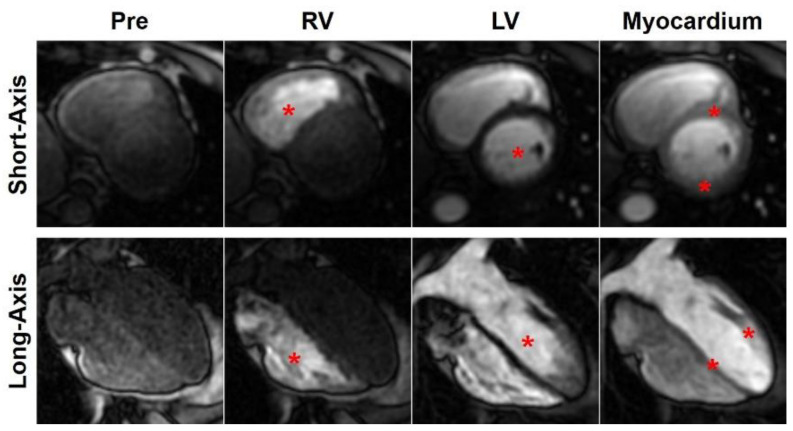
Myocardial perfusion imaging. Short-axis (**top**) and long-axis (**bottom**) series of T1-weighted saturation-recovery perfusion images acquired before contrast injection (pre), and at times of bolus arrival in the right-ventricular (RV) blood pool, left-ventricular (LV) blood pool, and myocardium, as denoted by the red asterisks. The images show normal perfusion without any defects.

In less than a minute after coronary artery occlusion, dysfunction of myocardial relaxation, wall motion abnormalities, and a decline in LV EF can occur before changes in cardiac conduction or angina are detected. Furthermore, acute coronary syndrome diastolic dysfunction predicts an increased potential for progression to myocardial infarction in the absence of ECG changes or cardiac enzyme elevation [90]. With this in mind, recent trends in research have shifted from traditional methods of acute coronary syndrome diagnosis to investigations using CT and MRI. For instance, Azarisman et al. [86] showed that acute coronary syndrome can be accurately and reliably diagnosed using different cardiac MRI sequences such as cine imaging, T2-weighted imaging, trans-mitral flow velocity imaging, first-pass myocardial perfusion (Figure 5), and delayed gadolinium enhancement, in the same amount of time it takes to obtain cardiac enzyme laboratory results. Thus, cardiac MRI has the potential to provide an accurate diagnosis of acute coronary syndrome while simultaneously risk-stratifying patients.

### 6.2. Myocardial Infarction

The assessment of global LV systolic function after acute myocardial infarction (AMI) has traditionally held important clinical and prognostic significance. For instance, previous studies using echocardiography have shown that patients with global diastolic dysfunction post-AMI have a worse prognosis than those without diastolic impairment [89]. It has also been demonstrated that distinctive valuable diagnostic and prognostic information relating to ischemic injury after AMI can be provided by regional functional evaluation [91]. The regional heterogeneity of diastolic dysfunction caused by the non-uniformity of ischemic events is best analyzed by cardiac MRI tagging. Correspondingly, results reported by Azevedo et al. [91] demonstrated significant impairment of systolic and diastolic regional function in both transmural and subendocardial regions using tagged MRI. Conversely, reversibly injured regions can demonstrate persistent diastolic dysfunction despite complete systolic functional recovery after re-perfused AMI. Additionally, the presence of microvascular obstruction was shown to play a role in regional diastolic impairment quantified using strain rate analysis of tagged MRI images [91]. The non-uniformity, delay, and prolongation of myocardial untwisting during the relaxation phase allowed for detecting prior anterolateral infarction [18], before reversal of ischemia (where possible) in hibernating myocardium [92], and ST-elevated myocardial infarction [93]. Furthermore, cardiac MRI velocity encoding allowed for detecting substantial impairment of regional, early diastolic, long-axis myocardial velocities, and LV filling in patients who suffer from angina and prior MI [20].

## 7. The Right Ventricle

### 7.1. Pulmonary Hypertension

**Figure 6:**
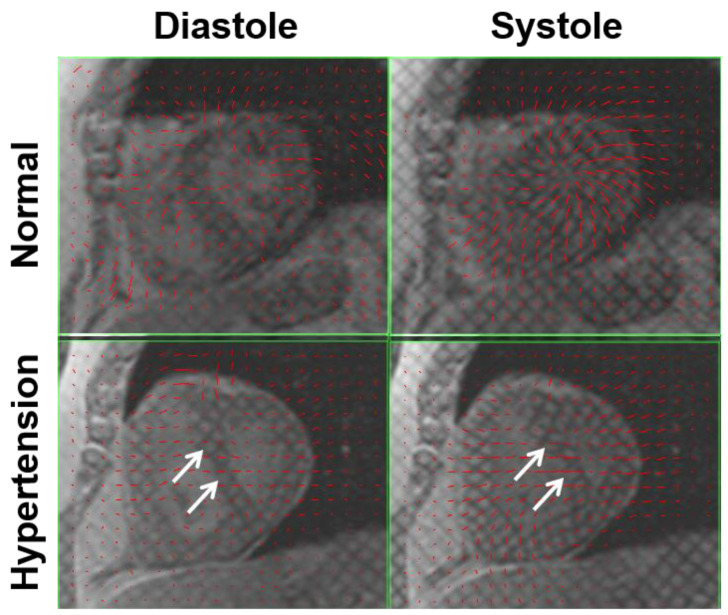
Left-ward septal wall bowing in pulmonary hypertension. Tagged images acquired in a normal case (**top**) and in pulmonary hypertension (**bottom**) during diastole (**left**) and systole (**right**). Note abnormal left-ward septal wall bowing (white arrows), especially during systole, in pulmonary hypertension due to increased pressure in the right ventricle. Displacement vectors calculated from the tagged images are represented by red arrows, which show reduced myocardial contractility in pulmonary hypertension.

RV diastolic function has also been evaluated using MRI. Tello et al. [94] were the first to delineate the pathology associated with RV strain in pulmonary hypertension (Figure 6) through comparison of MRI parameters with pressure-volume/Swan-Ganz catherization. Their results provided strong evidence supporting MRI RV strain as an indicator for RV-arterial uncoupling and diastolic stiffness, which gave insight into the relationship between RV contractility and afterload, changes in RV relaxation, and ventricular remodeling.

### 7.2. Chronic Obstructive Pulmonary Disease

RV dysfunction can be linked to LV dysfunction. LV hypertrophy and stiffness are common consequences of chronic obstructive pulmonary disease (COPD), which often has a negative impact on survival. Air trapping, pulmonary hypertension, and the resultant increase in RV volume overload are thought to be the cause of LV diastolic dysfunction in patients with COPD [95]. This ventricular interdependency is known to result from COPD and many cardiovascular diseases. Schafer et al. [52] recently reported correlations between RV diastolic and systolic functions with LV vorticity, suggesting early development of ventricular interdependency in COPD. Using 4D-Flow cardiac MRI, they identified changes in vortex formation and reduced vorticity in mild-to-moderate COPD, which possibly identify early changes in LV architecture due to lung over-inflation and the resultant physiologic changes in the intrathoracic cavity [52].

### 7.3. Tetralogy of Fallot

**Figure 7:**
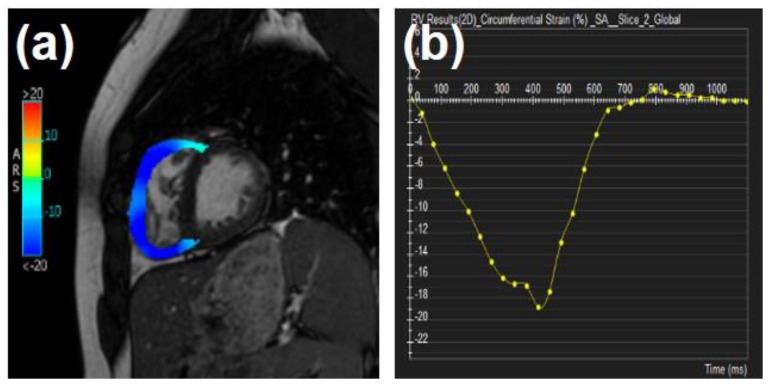
Tissue-tracking strain analysis in the right ventricle (RV) in tetralogy of Fallot (ToF). (**a**) RV circumferential strain map showing reduced strain at the RV insertion points. (**b**) Global circumferential strain curve in the RV.

MRI has also demonstrated utility in congenital heart disease, e.g., in the tetralogy of Fallot (TOF) (Figure 7). Together, velocity mapping and tomographic MRI provide a clinically practical way to quantify RV diastolic function and size and pulmonary regurgitation; thus allowing for adequate monitoring and longitudinal care of patients with surgical repair of TOF, something which cannot be done using Doppler echocardiography. In their study, Helbing et al. [24] found pulmonary regurgitation and RV diastolic dysfunction consistent with impaired relaxation in a cohort of young TOF patients, which could be responsible for their impaired exercise performance. However, Gatzoulis et al. [96] suggest the possibility that the myocardial restrictive process could be a limiting factor of pulmonary regurgitation in TOF, as the degree of pulmonary regurgitation did not increase relative to the degree of RV restriction as found by Helbing et al. [24]. In other congenital heart diseases, such as tricuspid atresia, 3D cardiac MRI tagging showed to be useful in the evaluation of ventricular diastolic dysfunction after the Fontan procedure by demonstrating anomalies of regional strain and reduced twist angle during systole [27].

## 8. Other Related Heart Diseases

### 8.1. Aortic Stenosis

Stuber et al. [15] showed a decrease in apex untwisting angle during the onset of diastole in patients with aortic stenosis (AS) when compared to normal controls or athletes with comparable amounts of physiologic hypertrophy. Another study demonstrated decreased basal rotation and ventricular twisting rate and increased apical rotation and ventricular torsion in patients with severe AS [18]. In their study, Uddin et al. [38] used CSPAMM to investigate changes as a result of AS repair by transcatheter aortic valve implantation (TAVI). They concluded that AS repair by TAVI led to improved mid-LV circumferential strain and reduced torsion, but failed to demonstrate changes in diastolic strain rate.

### 8.2. Hypertrophy

**Figure 8:**
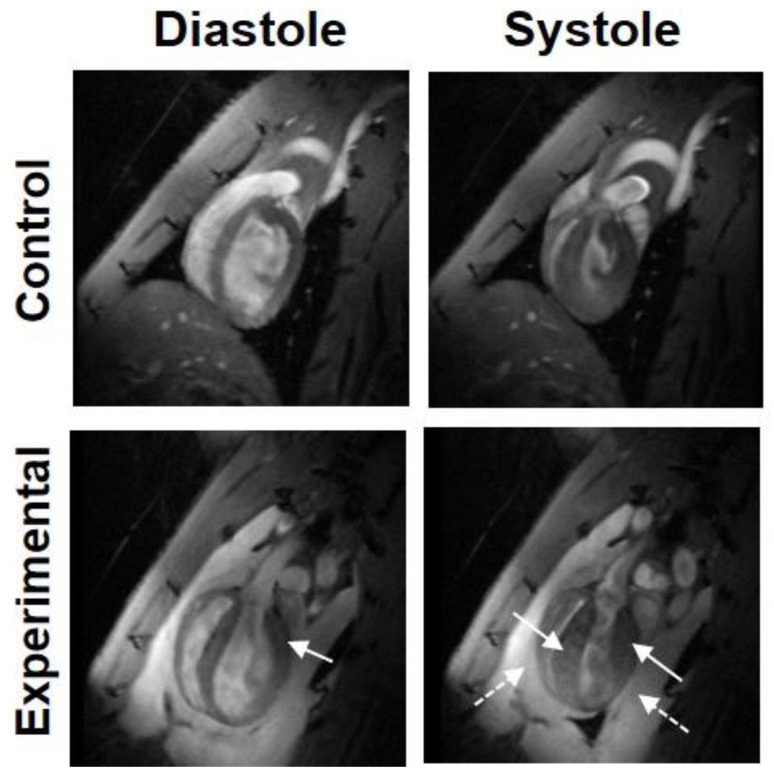
Myocardial hypertrophy in a rat model of lung cancer radiation therapy (experimental). Four-chamber cine images acquired during diastole (**left**) and systole (**right**) in a normal rat (**top**) and an experimental rat (**bottom**) that received localized heart irradiation of 24Gy. Note developed hypertrophy (solid arrows) and pleural effusion (dotted arrows) post radiation.

Pathophysiological changes such as sarcomere proliferation and myocardial wall stiffening as a result of pressure overload may explain the differences in rotation angle and ventricular filling pattern that is seen in pressure overload hypertrophy (Figure 8) [97]. Chacko et al. [98] showed that the time to early-peak filling rate was significantly prolonged in hypertrophic cardiomyopathy and hypertensive heart disease when compared to controls. They also showed that maximum LV wall thickness and time-to-early-peak filling rate correlated positively in those same groups, most notably in hypertensive heart disease, although maximum LV wall thickness was significantly higher in hypertrophic cardiomyopathy.

Li et al. [99] showed that LV outflow tract obstruction may result in increased LV wall stress, myocyte death, and fibrosis. As a result of their investigations, they showed that global peak diastolic strain rates were reduced in all planes in patients who had LV outflow tract obstruction as a result of hypertrophic cardiomyopathy. Additionally, patients who suffered major adverse cardiovascular events (MACE) were found to have a lower LV global peak diastolic strain rate. Cine MRI allowed for detecting changes correlating to hypertrophy, including impaired relaxation, as measured by prolongation of the time-to-peak rapid filling rate and the time-to-peak wall thinning rate, before mitral flow changes could be seen by Doppler imaging [17]. ^31^P-MRS predicted hypertrophic changes before they happen, possibly due to the lower myocardial phosphocreatine levels leading to calcium overload and impairment of cardiac cells [19]; furthermore, the decreased phosphocreatine to adenosine triphosphate (PCr/ATP) ratio correlated with the level of hypertrophy. MRI strain imaging offered the ability to differentiate the various types of the disease [13]. Russel et al. [100] showed that patients who were mutant carriers of familial hypertrophic cardiomyopathy had normal wall thickness but increased LV torsion in comparison to normal controls, which indicated that early intervention could protect against later cardiac dysfunction. The results from Svalgring et al. [44] demonstrated that 4D-Flow MRI is useful for the detection of abnormal flow patterns correlating with mild LV hypertrophy in patients with minimal or subclinical LV dysfunction.

### 8.3. Thalassemia

The risk for diastolic dysfunction is known to increase with age, obesity, and hypertension in the general population [33], but the determinants of increased risk for LV diastolic dysfunction among those with thalassemia major are not outlined well. It is known that homozygous beta-thalassemia leads to secondary hemochromatosis and iron overload cardiomyopathy, which is the leading cause of death in this population. In this respect, cardiac MRI T2* imaging is the current method of diagnosis for iron overload cardiomyopathy. In their study, Chinprateep et al. [101] determined that LV diastolic dysfunction can be detected by PC-MRI before systolic dysfunction occurs or signs of iron overload can be identified by T2* MRI. LV diastolic dysfunction could be explained by changes such as smooth muscle proliferation and vasoconstriction related to splenectomy, which is performed more frequently in beta-thalassemia major [101].

### 8.4. Pericarditis

Constrictive pericarditis is another cause of diastolic dysfunction and is reflected by the abrupt displacement of the interventricular septum in the LV during diastole. Alternatively, aberrant septal systolic motion into the RV is a common finding following cardiac surgery in constrictive pericarditis [102]. In their work, Spottiswoode et al. [103] showed that DENSE MRI is superior to cine imaging for determining the dominant postoperative systolic septal wall motion abnormality in pericarditis. Although in the case of tuberculous pericarditis treated with pericardiectomy, cine MRI conducted post-operation showed diastolic septal bounce of constriction in a mid-ventricular short-axis view, the images did not clearly demonstrate the septal bulging into the RV cavity during systole.

### 8.5. Cardiotoxicity

**Figure 9:**
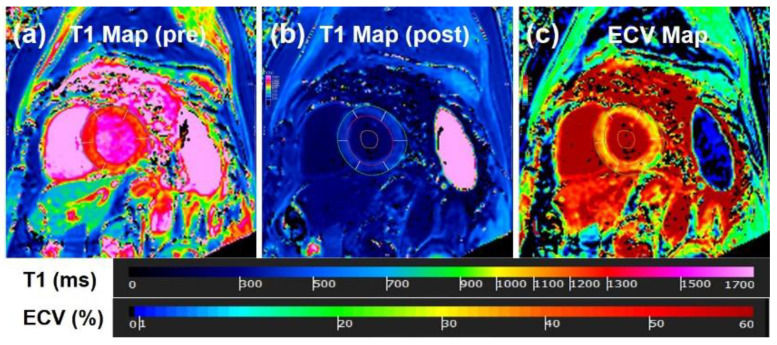
T1 mapping. (**a**) Pre-contrast T1 map, (**b**) post-contrast T1 map, and (**c**) generated extracellular volume (ECV) map. Higher ECV values reflect increased fibrosis or collagen formation.

**Figure 10:**
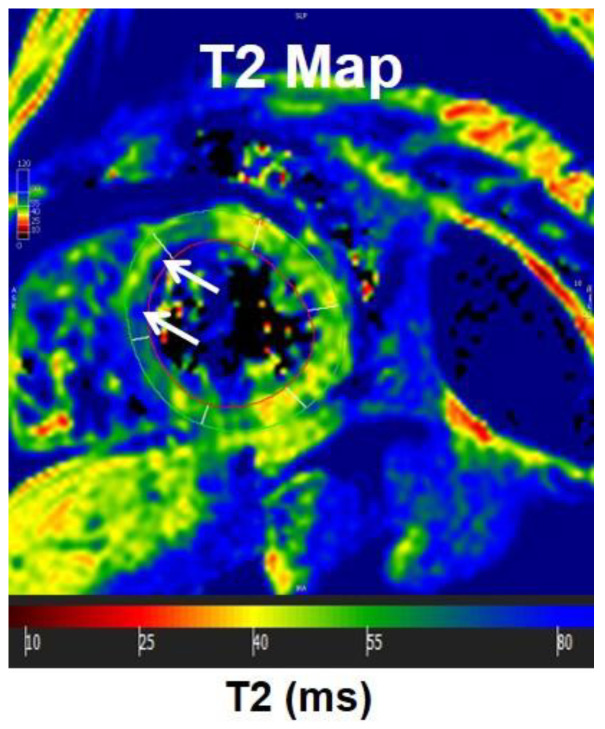
T2 mapping. T2 map in a mid-ventricular short-axis slice, showing increased T2 values in the anteroseptal wall (arrows). Increased T2 values could reflect edema.

Cancer treatment is known to have adverse cardiac effects on both systolic and diastolic functions and tissue composition (Figure 9 and Figure 10). Deleterious effects can vary from a mild decline in LV EF to severe HF, are not dose related, typically subclinical, and thought to be reversible [104]. Song et al. [105] reported data on subclinical diastolic LV dysfunction in relationship to LV systolic function as well as changes in volume during and after treatment of breast cancer with trastuzumab. Their study concluded that while deficiencies in LV systolic function were reversible, diastolic deficiencies, specifically the ratio of peak filling rate to LV EDV, persisted 18 months post treatment. Furthermore, unlike in other conditions such as anthracycline-induced cardiotoxicity and aging in which the development of diastolic dysfunction may occur before or in parallel with systolic dysfunction [106], the changes in LV systolic and diastolic function and volumes occur simultaneously during trastuzumab treatment [105]. Recently, Gong et al. [107] studied a cohort of breast cancer patients treated with trastuzumab over four years in order to investigate early-diastolic strain rate parameters measured by MRI-FT and their relation to subclinical trastuzumab induced cardiotoxicity. The results did not show a consistent correlation between early-diastolic strain rates and systolic strain rates or even consistent longitudinal variations of early-diastolic strain rates. The results also did not demonstrate a strong association of diastolic strain measurements with LV EF. Nevertheless, these findings are contradictory with previous results [108] as to whether a reduction in LV EDV is to blame for reduced systolic strain, or whether diastolic longitudinal strain rate was predictive of deteriorating LV function, which may actually be explained by differences in cohort demographics or analytical methods between the two studies.

### 8.6. Cardiac Allograft

The precise mechanism leading to the development of cardiac allograft vasculopathy is still unclear. Some suggestions include stiffness of the transplanted heart due to hypertrophy of cardiac myocytes and endocardial fibrosis, immunologic mechanisms combined with other risk factors for vascular injury, and the combination of systemic hypertension and microvascular stenosis [109]. Given that LV diastolic dysfunction is more sensitive to subendocardial ischemia than systolic dysfunction [110], it makes sense that peak flow rate measurement by MRI could be an adequate method for the early detection of cardiac allograft vasculopathy. Machida et al. [109] employed MRI measurement of peak flow rate to detect diastolic dysfunction, and thus, allow for early detection of cardiac allograft vasculopathy in heart transplant recipients.

## 9. Summary

MRI is gaining ground as a routine clinical tool for the evaluation of diastolic HF. Multiple cardiac MRI techniques have demonstrated superiority over methods that are currently considered the gold standard or are most commonly used. Furthermore, new methods for applying these techniques are being developed to eliminate time-consuming post processing and burdensome image acquisition. Armed with these advances, MRI is becoming a feasible and convenient tool for the accurate prediction, diagnosis, and long-term monitoring of diastolic dysfunction. It should be noted that although cardiac MRI provides several parameters that allow for evaluating heart morphology and function, myocardial contractility and tissue characterization, and hemodynamic parameters, its clinical utility could be affected by its relatively high cost, long scan time, and the need for experienced operators. Nevertheless, recent advances in MRI hardware, pulse sequences, and artificial intelligence (AI) capabilities allow for mitigating these limitations. For example, phased-array coils allow for parallel imaging capabilities that reduce scan time [111]. Furthermore, recently introduced commercial pulse sequences allow for single-heartbeat and real-time cardiac imaging with the capability of completing a comprehensive cardiac MRI exam in a short time, which results in reducing scan cost and making cardiac MRI available to more patients [112]. Similarly, 3D imaging sequences allow for reduced scan time and simplified scanner operation, where the operator needs only to place a box around the heart without the need for a cardiac trained operator or a physician to attend the scan [112,113]. Finally, recently introduced AI-supported MRI techniques allow for automatic parameter settings based on patient-specific conditions (e.g., heart rate, respiratory pattern, and breath-holding capability) as well as AI-supported image reconstruction which reduces acquisition time and improves image quality [114]. In summary, recent advancements in MRI technology are expected to result in wider clinical adoption of cardiac MRI in clinical practice and more availability in community hospitals and for larger patient populations.

## Figures and Tables

**Table 1:** MRI and diastolic function: applications in cardiac disease.

Type of Disease	MRI Technique	*n*	Principal Findings
HCM [11]	GRE	31	Impairment of regional relaxation
HCM [12]	Spectroscopy	8	Decreased PCr/ATP in symptomatic patients
HCM [13]	Spectroscopy	8	Decreased PCr/ATP in asymptomatic patients
HCM [14]	Tagging	17	Smaller circumferential curvatures in hypertrophy
AS [15]	Tagging	12	Prolonged and delayed untwisting
AS [16]	Phase contrast	9	Volumetric mitral flow correlates with Doppler
LVH [17]	GRE	9	Early detection of filling abnormalities
AS [18]	Tagging	13	Prolonged and delayed untwisting
Hypertensive HD [19]	Spectroscopy	11	Decreased PCr/ATP correlates with impaired relaxation
Previous MI [20]	Phase contrast	11	Early diastolic filling velocities correlate with Doppler
Previous MI [21]	Tagging	16	Reduction of systolic strains in infarcted and remote area
Previous MI [22]	Tagging	18	Nonuniform, delayed, and prolonged untwisting
CAD/previous MI [20]	GRE	10/15	Reduced early diastolic long-axis velocity
Previous MI [23]	Tagging	9	Reduced systolic strains in asynergic segments
Fallot [24]	Phase contrast	19	Restrictive flow is associated with decreased exercise
Mustard/Senning [25]	Phase contrast	12	Restrictive tricuspid flow
Fallot [26]	GRE	10	Impaired ventricular filling correlates with exercise
RVPO [14]	Tagging	9	Heterogeneity in strain
Single ventricle [27]	Tagging	10	Regional decrease in systolic strains

AS, Aortic stenosis; ATP, adenosine triphosphate; CAD, coronary artery disease; GRE, gradient echo; HCM, hypertrophic cardiomyopathy; HD, heart disease; LVH, left ventricular hypertrophy; MI, myocardial infarction; PCr, phosphocreatine; RVPO, right ventricular pressure overload; Spectroscopy, ^31^P-MR spectroscopy. Table reproduced with permission from [10]. Copyright 2002 Elsevier.

**Table 2:** Ventricular parameters of diastolic function.

Class of Diastolic Dysfunction	HC	I	II	III	*p*
*n*	25	18	12	10	
LV filling volume (mL)	69 ± 16	66 ± 17	61 ± 23	69 ± 17	0.72
PFR-E (mL/s)	375 ± 63 ^I,II^	247 ± 47 ^H,II,III^	325 ± 47 ^H,I^	353 ± 92 ^I^	0.001
PFR-E/LV filling volume (s)	5.4 ± 1.3 ^I^	4 ± 0.8 ^H,III^	4.7 ± 2	5.4 ± 1.3	0.02
PFR-A (mL/s)	177 ± 56 ^I,III^	238 ± 59 ^H,III^	209 ± 83 ^III^	136 ± 37 ^H,I,II^	0.02
PFR-A/LV filling volume (s)	2.3 ± 1.1 ^I,II^	3.9 ± 1.2 ^H,II,III^	1.5 ± 0.8 ^H,I,III^	2.6 ± 1.6 ^H,I,II^	0.001
PFR-E/PFR-A	2.3 ± 1 ^I^	1.1 ± 0.4 ^H,II,III^	2.1 ± 1 ^I^	2.8 ± 1.3 ^I^	0.001

*p* values were derived by ANOVA testing with Bonferroni correction for multiple comparisons. The ANOVA test was also used for single comparison between groups: ^H^ significant difference vs. healthy controls (*p* < 0.05); ^I^ significant difference vs. grade I diastolic dysfunction (*p* < 0.05); ^II^ significant difference vs. grade II (*p* < 0.05); ^III^ significant difference vs. grade III (*p* < 0.05). LV left ventricular, PFR-A atrial peak filling rate, PFR-E early peak filling rate. Table reproduced with permission from [56]. Copyright 2018 Springer Nature.
